# Characterization and Optimization of the Tyrosinase Inhibitory Activity of *Vitis amurensis* Root Using LC-Q-TOF-MS Coupled with a Bioassay and Response Surface Methodology

**DOI:** 10.3390/molecules26020446

**Published:** 2021-01-16

**Authors:** Kyung-Eon Oh, Hyeji Shin, Mi Kyeong Lee, Byoungduck Park, Ki Yong Lee

**Affiliations:** 1 College of Pharmacy, Korea University, Sejong 30019, Korea; ok1000sa@korea.ac.kr (K.-E.O.); hjshin90@korea.ac.kr (H.S.); 2 College of Pharmacy, Chungbuk National University, Cheongju 28160, Korea; mklee@chungbuk.ac.kr; 3 College of Pharmacy, Keimyung University, Daegu 42403, Korea

**Keywords:** *Vitis amurensis*, LC-Q-TOF-MS coupled with tyrosinase inhibitory assay, response surface methodology, Pearson’s correlation

## Abstract

*Vitis amurensis* roots have been reported to have the potential for skin whitening through the evaluation of melanogenesis and tyrosinase inhibitory activities. In this study, *V. amurensis* roots were utilized to quickly select whitening ingredients using LC-Q-TOF-MS coupled with tyrosinase inhibitory assay, and to optimize the extraction process for use as a skin whitening functional material by response surface methodology. Results showed that *V. amurensis* roots exhibited tyrosinase inhibitory effects by two stilbene oligomers, ε-viniferin (**1**) and vitisin B (**2**), as predicted by LC-Q-TOF-MS coupled with bioassay. The optimal extraction conditions (methanol concentration 66%, solvent volume 140 mL, and extraction time 100 min) for skin whitening ingredients were established with the yields 6.20%, and tyrosinase inhibitory activity was 87.27%. The relationship between each factor and its corresponding response was confirmed by Pearson’s correlation analysis. The solvent volume showed clear linear relationship with yields, and methanol concentration had a strong linear relationship with tyrosinase inhibitory activity for compounds **1** and **2**, as well as their combination. Overall, LC-Q-TOF-MS coupled with bioassay was proved to have the potential to effectively find new active constituents, as well as known active constituents; vitisin B can be proposed as a new natural potential whitening agent.

## 1. Introduction

Melanin is responsible for the color of mammalian skin and hair and protects the skin from ultraviolet rays, but excessive melanin production and accumulation of melanin in the skin cause hyperpigmentation skin disorders such as freckles, melasma, age-spots, ephelides, and senile lentigines. Tyrosinase, known as a copper-containing oxidase enzyme, has a crucial role in melanin biosynthesis. The enzyme catalyzed two consecutive oxidation reactions: The first step, the hydroxylation of l-tyrosine to 3,4-dihydroxy-l-phenylalanine (l-DOPA), and the second step, the oxidation of the l-DOPA to dopaquinone. Dopaquinone is a highly reactive substance that can polymerize spontaneously to generate melanin [1,2,3]. Hence, tyrosinase inhibitors can be used as treatments for hyperpigmentation-related skin disorders and as skin-whitening agents.

*Vitis amurensis*, a wild-growing grape species, is mainly distributed in Asia (Korea, China, and Japan). The fully ripe fruits are consumed raw and contain abundant nutrients such as sucrose, glucose, protein, and vitamins, so they are used as a material for wine, juice, jellies, and jam. Additionally, its leaves are used in a salad [4]. Its roots and stems have been used as traditional medicinal for the treatment of cancer, neuralgic pain, and abdominal pain [5,6]. Its roots consist of stilbenes (main constituent), procyanidins, flavonoids, triterpenoids, and other phenolic compounds. Until now, chemical compositions of the root have been studied on sufficient detail. In particular, various stilbene oligomers, including a resveratrol, amurensin A, vitisin A, (+)-ε-viniferin, amurensins C–M, ampelopsin A, D, and ampelopsin E, were reported [6]. The methanolic extract of the root exhibits anti-melanogenic effect against α-melanocyte stimulating hormone-induced melanogenesis in B16F10 cells and in 3,4-dihydroxyphenylalanine (L-DOPA) oxidation via mushroom tyrosinase [7]. Additionally, *V. amurensis* extracts and its active compounds exhibit antioxidant, anti-inflammatory, neuroprotective, and anti-tumorigenic effects [7,8,9,10].

LC-MS combined with a bioassay can simultaneously confirm the chemical profile and biological activity of components in natural products without the need for extraction and isolation. Therefore, it has recently been used to efficiently and quickly identify bioactive compounds in natural products [11,12,13].

Optimization is a process that allows for the maximum efficiency of experimental systems or products. Response surface methodology (RSM), multivariate analysis, design of experiments using mathematical and statistical techniques based on empirical models, and expression of the correlation between experimental design and results as a polynomial function, are some techniques that provide ideal optimization conditions with maximum efficiency. RSM is an accurate and efficient optimization method widely used in various fields, including food processing, chemistry, biology, and agriculture [14,15,16]. Over the past decades, the interest in pharmaceuticals, cosmetics, and functional foods containing natural products has increased; consequently, there is ongoing research in both academia and industry aimed at developing such products [17,18]. The first step in these studies involves extraction of the bioactive constituent from natural products. At this time, since numerous factors such as extraction time, temperature, liquid-solid ratio, and solvent volume affected the extracted constituents, an optimization for extracting the bioactive constituents to the maximum is required.

To our knowledge, tyrosinase inhibitory constituents and optimization of the extracts of *V. amurensis* roots have rarely been reported [5,19]. This study therefore aimed to quickly obtain the tyrosinase inhibitor from *V. amurensis* roots using LC-Q-TOF-MS coupled with tyrosinase inhibitory assay and to optimize the extraction conditions for broadening the utilization of *V. amurensis* roots as a skin whitening agent by RSM.

## 2. Results and Discussion

### 2.1. LC-QTOF MS Coupled with a Tyrosinase Inhibitory Assay Using the Root Extract of V. amurensis

**Figure 1:**
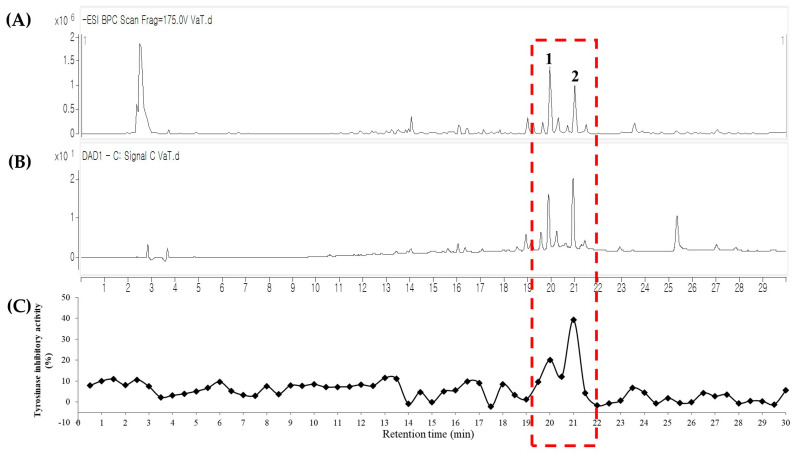
LC-QTOF-MS coupled with a tyrosinase inhibitory assay of extraction of *V. amurensis* root. (**A**) MS chromatogram in negative ionization mode; (**B**) UV chromatogram at 280 nm; (**C**) tyrosinase inhibitory activity of 30 s interval eluents.

The 80% MeOH extract of *V. amurensis* root exhibited significant tyrosinase inhibitory activity (80.7 ± 0.8% at 50 µg/mL, Table S1). To identify the tyrosinase inhibitory compounds in *V. amurensis* root without isolation, LC-QTOF-MS coupled with a tyrosinase inhibitory assay was conducted. The chemical profile of the *V. amurensis* root extract was obtained in the first run (Figure S1), and the bioactive compounds were identified using a tyrosinase inhibitory assay of fractions collected every 30 *s* from the second run (Figure 1). There were two peaks between 19 and 22 min on the mass chromatogram predicted to have significant tyrosinase inhibitory activity, and their structures were identified to be stilbene dimer (**1**) and stilbene tetramer (**2**) using chemical profiling (Table 1).

### 2.2. Identification of Tyrosinase Inhibitory Constituents of V. amurensis Root

**Figure 2:**
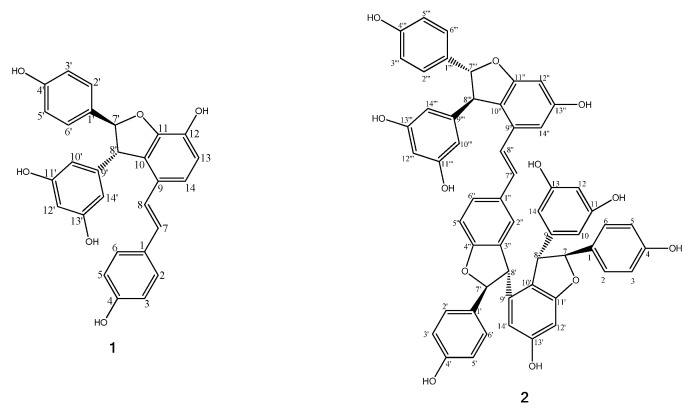
Chemical structures of ε-viniferin (**1**) and vitisin B (**2**).

First, two constituents expected to have tyrosinase inhibitory activity were isolated from the EtOAc fraction and their bioactivity evaluated. The structures of the isolated compounds **1** and **2** were identified as ε-viniferin (**1**) [20,21] and vitisin B (γ-viniferin, **2**) [21,22,23], respectively, using ^1^H-NMR, ^13^C-NMR, and ESI-MS (Figure 2, Figures S2 and S3, and Table S2). In tyrosinase inhibitory assay, the IC_50_ values of compounds **1**, **2**, and kojic acid were 3.51, 10.74, and 27.09 µM, respectively. Both compounds showed higher tyrosinase inhibitory effects than the positive control, kojic acid which is the known skin whitening constituent (Table 1 and Figure S4). In previous studies, ε-viniferin (**1**) is reported to have tyrosinase inhibitory activity [23]; however, vitisin B (**2**) was first identified in our study.

**Figure 3:**
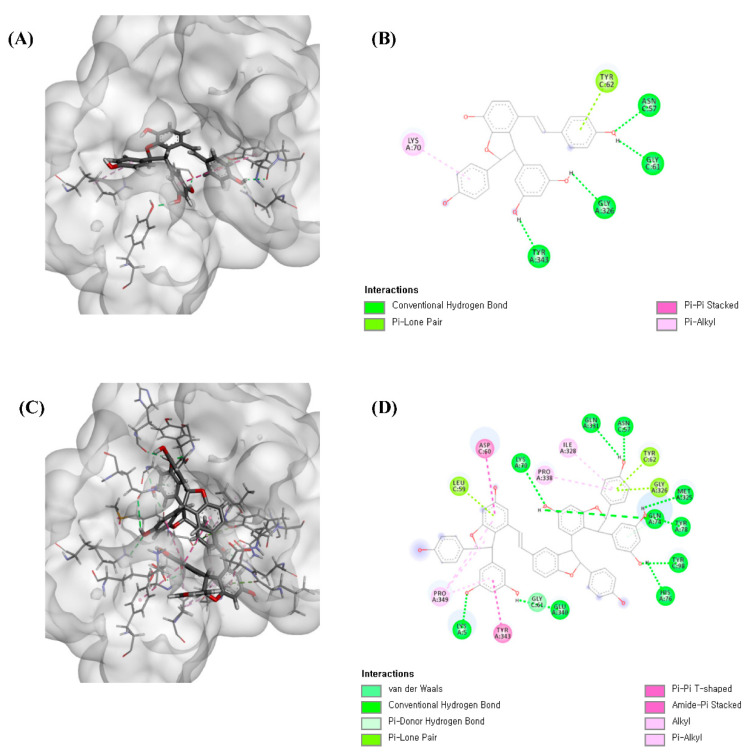
(**A**) 3D docking picture of compound **1**; (**B**) 2D docking picture of compound **1**; (**C**) 3D docking picture of compound **2**; (**D**) 2D docking picture of compound **2**.

Overall, the results, correlated with the predicted data of LC-MS coupled with a tyrosinase inhibitory assay and vitisin B (**2**), show potential as a new tyrosinase inhibitor. Furthermore, molecular docking studies were conducted to support the result of the significant tyrosinase inhibitory activity of the two stilbene oligomers. As shown in Table 1, compounds **1** and **2** showed a higher docking score than the positive control, kojic acid, consistent with our experimental data. However, the docking results of compounds were contrary to our experimental results. The interaction modes of compounds **1** and **2** were described in Figure 3. Compound **1** formed 4 hydrogen bonds, 2 hydrophobic interactions, and 1 pi-lone pair interaction, and compound **2** formed 11 hydrogen bonds, 7 hydrophobic interactions, 1 van der Waals interaction, and 3 pi-lone pair interactions. As a result, it was confirmed that compounds can be inserted into the active site of the target protein and bind to catalytic amino acid residues that can inhibit tyrosinase activity. Additionally, ε-viniferin (**1**) is reported as a competitive inhibitor that binds to the same site that L-DOPA binds to tyrosinase [23]. It was observed that vitisin B (**2**) binds to the same site as ε-viniferin (**1**), which confirmed that vitisin B (**2**) is a new competitive inhibitor.

### 2.3. Optimization of V. amurensis Root Extraction Using RSM

In order to utilize the *V. amurensis* roots as a skin whitening functional material, the optimal extraction conditions were designed by Box-Behnken design (BBD) to maximize extraction yield and tyrosinase inhibitory activity. The effects of independent responses such as extraction yield, tyrosinase inhibitory activity, amount of compound **1**, amount of compound **2**, and amount of the sum of compounds **1** and **2,** on the three independent variables (extraction time, MeOH/water concentration, and solvent volume), were measured (Table 2). The range of variables was set as extraction time (40, 70, and 100 min), MeOH concentration (40, 70, and 100%), and solvent volume (35, 87.5, and 140 mL) based on a preliminary single-factor experiment (data not shown). The values obtained from the designed experiments were expressed as polynomials of correlations between variables using regression analysis (Tables S4–S13 and Figure S5). As a result of performing individual optimization for each reaction (Table 3), the yield was expected to represent 6.21% when extracted with 100.00 min, MeOH 64.78%, 140.00 mL. Tyrosinase inhibitory activity (%) was extracted with 65.22 min, MeOH 100.00%, 140.00 mL conditions, and it was predicted to show a value of 90.37%. Amount of compound **1** at 65.74 min, MeOH 100.00%, 35.00 mL was predicted as 37.45 µg/mg, and amount of compound **2** at 70.00 min, MeOH 70.00%, and 92.24 mL was predicted as 86.77 µg/mg. In addition, the total contents of compounds **1** and **2** were expected to show a maximum value of 108.10 µg/mg when extracted under conditions of 75.20 min, MeOH 100.00%, and 35.00 mL. Experiments based on optimized conditions yielded 6.19 ± 0.36%, tyrosinase inhibitory activity 91.72 ± 3.48%, compound **1** content 36.54 ± 1.78 µg/mg, compound **2** content 85.74 ± 16.57 µg/mg, and sum of compounds **1** and **2** 108.10 ± 19.11 µg/mg were obtained, and individual responses for each variable exhibited a difference of 5% or less from the theoretical predicted values. Multiple response optimization was performed to maximize extraction yield and tyrosinase inhibitory activity (Table 3). The optimized conditions were as follows: extraction time, 100 min; MeOH concentration, 66.38%; and solvent volume, 140 mL. Using these conditions, the yield was determined to be 5.95 ± 1.13% and the tyrosinase inhibitory activity was 85.93 ± 1.57%; these values were similar to the predicted values, 6.20 and 87.25%, respectively. Additionally, the correlation between each variable and the corresponding response was analyzed using Pearson’s correlation (Table 4). The extraction yield showed a clear linear relationship between the extraction time and MeOH concentration and a negative linear relationship with amount of compound **1**. Additionally, the tyrosinase inhibitory activity showed a strong linear relationship between amount of compound **2** and amount of the sum of compounds **1** and **2**, and a clear linear relationship with compound **1**. Therefore, the tyrosinase inhibitory activity of *V. amurensis* root was proportional to both compounds **1** and **2**, but exhibited a stronger linear relationship with the amount of compound **2** than compound **1**.

## 3. Materials and Methods

### 3.1. General Experimental Procedures

Medium pressure liquid chromatography (MPLC) was conducted using a Biotage Isolera (Biotage AB, Uppsala, Sweden). One system equipped with a high performance flash chromatography (HPFC) pump, a variable dual-wavelength detector, and a collector. NMR spectra were acquired using a Bruker SPECTROSPIN 300 MHz spectrometer (Bruker Corporation, Billerica, MA, USA). Methanol-d_4_, an NMR solvent, was purchased from Cambridge Isotope Laboratories, Inc. Acetonitrile (ACN), water, and methanol (MeOH) of chromatographic grade were purchased from ThermoFisher Scientific Korea Ltd. (Seoul, Republic of Korea). L-tyrosine, mushroom tyrosinase, kojic acid, and formic acid were purchased from Sigma–Aldrich Co (St. Louis, MO, USA).

### 3.2. Plant Material

*V. amurensis* root was obtained from Gyeongbuk, Korea, and also purchased from Omniherb (Daegu, Republic of Korea). They were identified by Dr. Prof. Ki Yong Lee, from the College of Pharmacy at Korea University. A voucher specimen (KUP-HD071) was deposited at the Laboratory of Pharmacognosy, College of Pharmacy, Korea University.

### 3.3. LC-Q-TOF Mass Spectrometry

LC was performed using an Agilent 1260 series (Agilent, Santa Clara, CA, USA) comprising a binary pump, online degasser, auto sampler, thermostatically controlled column compartment, and photodiode array detector. Chromatographic separation was performed using a Shiseido CapCell PAK C18 column (5 µm, 4.6 mm, I.D × 150 nm). The mobile phase consisted of water (solvent A) and ACN (solvent B), both containing 0.1% formic acid. The gradient conditions were as follows: 0–5 min, 10% B, 5–30 min, and linearly increase B from 10 to 90%. The flow rate was set to 0.6 mL/min; 5 µL and 20 µL of the samples were injected for LC-Q-TOF-MS analysis and LC-Q-TOF-MS coupled with tyrosinase inhibitory assay, respectively. Mass spectrometry was performed using an Agilent 6530 Q-TOF mass spectrometer (Agilent, Santa Clara, CA, USA) with an electrospray ionization (ESI) interface in negative mode. Data of mass range from *m*/*z* 50–1000 was collected in centroid mode. The mass parameters were as follows: capillary voltage, 4000 V; nebulizer pressure, 40 psi; fragmentor voltage, 175 V; skimmer voltage, 65 V; drying gas temperature, 325 °C; flow rate of drying gas, 12.0 L/min; collision energy 10, 20, 30, and 40 eV. Acquisition parameter adjustment and data processing were performed using LC-MS/MS Data Acquisition by using 6530 series Q-TOF (version B.05.00) (MassHunter Workstation software, Agilent, Santa Clara, CA, USA).

### 3.4. LC-Q-TOF-MS Coupled with a Tyrosinase Inhibitory Assay

LC-Q-TOF-MS coupled with a tyrosinase inhibitory assay was conducted using the method established in previously study [24]. In brief, the assay proceeded in two runs. In the first run, the chemical profile of the sample was obtained using LC-Q-TOF-MS. In the next run, the eluate after passing through the LC system under the stated LC-Q-TOF conditions was collected in 96-well plates every 30 s. The tyrosinase inhibitory activity of the collected fractions was evaluated using a tyrosinase inhibitory assay.

### 3.5. Isolation of Tyrosinase Inhibitory Compounds from V. amurensis Root

For the isolation of tyrosinase inhibitory compounds identified using LC-Q-TOF-MS coupled with the tyrosinase inhibitory assay, *V. amurensis* root (3.01 kg) was extracted three times with 80% MeOH for 60 min at room temperature using ultrasonication. The extracted solvent was filtered and concentrated to obtain a crude extract (215.7 g), which was suspended in water and sequentially partitioned using *n*-hexane, ethyl acetate (EtOAc), and *n*-BuOH. The EtOAc fraction (25.85 g) was subjected to silica gel column chromatography using *n*-hexane:EtOAc under gradient conditions (20:1 → 0:1) to yield seven fractions (E1–E7). Fraction E4 was separated using MPLC and 100 g SNAP KP-Sil, a silica gel cartridge, and dichloromethane:MeOH under gradient conditions (97:3 → 0:100) to yield seven sub-fractions (E4–1 to E4–7). Compound **2** (417.0 mg) was obtained from E4–5. Sub-fraction E4–4 was re-chromatographed on MPLC using SNAP 25 g Ultra, a silica gel cartridge, and chloroform:MeOH:H_2_O under gradient conditions (50:4:1 → 15:4:1) to yield seven fractions (E4–4–1 to E4–4–7). Compound **1** (396.0 mg) was obtained from E4–4–5, which was observed as a single spot on a thin layer chromatography (TLC) plate.

### 3.6. Tyrosinase Inhibitory Assay

The tyrosinase inhibitory activity was evaluated using a previously described method with slight modification [25]. The two microliters of sample and 50 µL of 0.1 U/µL mushroom tyrosinase were treated in 96-well plates and incubated at 37 °C. After 15 min, 50 µL of 1 mM L-tyrosine was added and then reacted at 37 °C for 15 min. The amount of dopachrome formed was measured at 495 nm using a Spectra Max 190 microplate reader (Molecular Devices, San Jose, CA, USA). Tyrosinase inhibitory activity was calculated using the following equation: $\mathrm{tyrosinase}\;\mathrm{inhibition}\;(\%)\;=\;\left[1-\left(S-S_{0}\right)/\left(C-C_{0}\right)\right]\times 100$, where S is the absorbance of sample, tyrosinase, and L-tyrosine; S_0_ is the absorbance of the sample and L-tyrosine; C is the absorbance of tyrosinase and L-tyrosine, and C_0_ is the absorbance of L-tyrosine. Kojic acid, a known tyrosinase inhibitor, was used as positive control. IC_50_ values were calculated using GraphPad Prism 6 (GraphPad Software, Inc., La Jolla, CA, USA).

### 3.7. Molecular Docking Studies

Molecular docking was performed by using SYBYL-X 2.1.1 software (Tripos Ltd., St. Louis, MO, USA) with crystal structures of PPO3, a tyrosinase from *Agaricus bisporus* (Protein Data Bank (PDB) ID: 2Y9W). All the water molecules of the target protein were removed, and ligand preparation was conducted by “sanitize” preparation protocol in SYBYL-X 2.1.1. Protein-ligand affinity was calculated by Tripos force field and expressed as total scores. The docked pose of ligand from the protein-ligand complex was visualized in Discovery Studio 2017 R2 Client program (Biovia Co., San Diego, CA, USA).

### 3.8. Experimental Design and Statistical Analysis

An optimized condition for extracting constituents with maximum tyrosinase inhibitory activity from *V. amurensis* root was established using the BBD with three variables and three levels (MINITAB Release 14.12.0 Statistical Software). Based on the preliminary single-factor experiment results, the independent variables including extraction time (X_1_), MeOH and water concentration (X_2_), and liquid volume (X_3_), and a range of their variables were selected (Table S3). The variables for RSM were coded using three levels, −1, 0, and 1. Overall, 15 experiments were designed including 3 replicates at the center of the design (Table 2). As independent responses, yield (%), tyrosinase inhibitory activity (%), the amount compound (**1**) (µg/mg), and the amount of compound (**2**) (µg/mg), were measured. Tyrosinase inhibitory activity of extract was evaluated at a concentration of 50 µg/mL. Each response is expressed using the following second-order polynomial equation:

$$R=\beta_{0}+\beta_{1}X_{1}+\beta_{2}X_{2}+\beta_{3}X_{3}+\beta_{12}X_{1}X_{2}+\beta_{23}X_{2}X_{3}+\beta_{13}X_{1}X_{3}+\beta_{11}X_{1}{}^{2}+\beta_{22}X_{2}{}^{2}+\beta_{33}X_{3}{}^{2},$$

where R denotes the response; β_1_, β_2_, and β_3_ are the linear coefficients; β_12_, β_23_, and β_13_ are the interaction coefficients between three variables; and β_11_, β_22_, and β_33_ are the quadratic coefficients.

Furthermore, Pearson’s correlation analysis was performed to determine the existence of a linear relationship between each variable and response. Pearson’s correlation coefficient has a strong linear relationship between 0.7 and 1.0, a clear linear relationship between 0.3 and 0.7, a weak linear relationship between 0.1 and 0.3, and no or negligible linear relationship between 0.0 and 0.1. The positive and negative correlation are expressed depending on whether the Pearson’s correlation coefficient is positive or negative.

### 3.9. Quantitative Analysis of Tyrosinase-Inhibitory Compounds ***1*** and ***2***

The amount of each compounds **1** and **2** in extracts obtained using the designed 15 experimental conditions were measured using the calibration curves (Table 2). The calibration curves for compounds **1** and **2** were determined using the area under the UV chromatogram curve (330 nm acquired at concentrations of 0.1–1000 µg/mL and 7.81–1000 µg/mL, respectively). LC was performed using a Waters 2695 LC system (Waters, Santa Clara, CA, USA) with the same conditions as those of the LC system detailed in Materials and Methods, LC-Q-TOF mass spectrometry.

## 4. Conclusions

ε-Viniferin (**1**) and vitisin B (**2**) of *V. amurensis* roots were characterized as skin whitening constituents using LC-Q-TOF-MS coupled with a tyrosinase inhibitory assay. In particular, vitisin B (**2**) was first identified as a tyrosinase inhibitory compound in this study and ε-viniferin (**1**) and vitisin B (**2**) showed higher tyrosinase inhibitory effects than the positive control, kojic acid. The optimization conditions with maximum tyrosinase inhibitory effect and yield of *V. amurensis* roots were established using extraction time (100 min), MeOH concentration (66.38%), and liquid volume (140 mL). The result exhibited a good correspondence between experimental and predicted values. Consequently, LC-Q-TOF-MS coupled with bioassay is proved the potential to effectively find new active constituents as well as known active constituents, vitisin B (**2**) can be proposed as a new natural potential whitening agent.

## Figures and Tables

**Table 1:** Chemical profiles of bioactive peaks detected using LC-QTOF coupled with a tyrosinase inhibitory assay.

Peak	Compound Identified	t_R_ (mins)	Observed *m*/*z*	Calculated *m*/*z*	Molecular Formula [M-H]^−^	MS/MS Fragments (*m*/*z*)	UV (λmax, nm)	IC_50_ (µM) ^a^	Total Score ^b^
**1**	ε-viniferin	19.837	453.1331	453.1344	C_28_H_21_O_6_	359[M-C_6_H_6_O-H], 347[M-C_7_H_6_O-H], 225[M-C_8_H_7_O-C_6_H_5_O-H]	284, 330	3.51 ± 0.1	8.5024
**2**	vitisin B	21.328	905.2894	905.2604	C_56_H_41_O_12_	811[M-C_6_H_6_O-H]	243, 313, 285	10.74 ± 1.3	9.5492

^a^ Half maximal inhibitory concentration (IC_50_) value of tyrosinase inhibitory activity. Positive control: kojic acid IC_50_ 27.09 ± 2.8 µM. ^b^ Molecular docking results showing the affinity between tyrosinase-compounds **1** and **2**. The total score of kojic acid: 6.0980.

**Table 2:** Responses for the Box-Behnken experimental design.

Run	Extraction Time (min)	MeOH Conc. (%)	Solvent Volume (mL)	Yield (%)	Tyrosinase Inhibitory Activity (%)	Compound 1 (µg/mg)	Compound 2 (µg/mg)	Sum of Compounds 1 and 2 (µg/mg)
1	40	40	87.5	2.60	49.79	−0.28	−4.15	−4.44
2	100	40	87.5	3.47	73.34	0.69	3.59	4.28
3	40	100	87.5	2.64	88.99	18.53	64.52	83.05
4	100	100	87.5	3.28	88.96	14.38	94.03	108.41
5	40	70	35	2.86	80.29	6.70	13.51	20.21
6	100	70	35	4.01	82.68	5.95	25.01	30.96
7	40	70	140	5.16	80.35	6.80	28.00	34.79
8	100	70	140	6.63	84.83	5.65	26.39	32.03
9	70	40	35	3.39	62.83	−0.05	−2.00	−2.05
10	70	100	35	2.51	88.60	43.39	64.17	107.56
11	70	40	140	4.77	71.82	2.72	13.96	16.67
12	70	100	140	3.47	89.88	11.32	78.64	89.96
13	70	70	87.5	5.06	91.08	12.82	97.69	110.51
14	70	70	87.5	4.18	88.40	12.23	82.92	95.15
15	70	70	87.5	4.24	87.63	11.34	78.91	90.24

**Table 3:** Predicted and experimental values of responses at optimized conditions.

Responses	Optimized Conditions			Composite Desirability (D)	Actual Values	Predicted Values	Predictive Capacity (%)
	Extraction Time (X_1_, min)	MeOH Concentration (X_2_, %)	Solvent Volume X_3_, mL)				
Yield (%)	100.00	64.78	140.00	0.94	6.19 ± 0.36	6.21	99.73
Tyrosinase inhibitory activity (%)	65.22	100.00	140.00	0.98	91.72 ± 3.48	90.37	101.50
Compound **1** (µg/mg)	65.74	100.00	35.00	0.91	36.54 ± 1.78	37.45	97.58
Compound **2** (µg/mg)	70.00	70.00	92.24	0.99	85.94 ± 16.57	86.77	99.05
Sum of compounds **1** and **2** (µg/mg)	75.20	100.00	35.00	0.98	108.10 ± 19.11	107.93	100.15
	**Multiple response optimization**						
Yield (%)	100.00	66.38	140.00	0.93	5.95 ± 1.13	6.20	96.03
Tyrosinase inhibitory activity (%)				0.91	85.93 ± 1.57	87.25	98.48

**Table 4:** Pearson’s correlation variables and responses.

	Extraction Time	MeOH Concentration	Solvent Volume	Yield	Tyrosinase Inhibitory Activity	Compound **1**	Compound **2**	Sum of Compounds **1** and **2**
Extraction Time								
MeOH concentration	0.000							
Solvent volume	0.000	0.000						
Yield	0.338	−0.191	0.594 *					
Tyrosinase inhibitory activity	0.245	0.794 ***	0.100	0.223				
Compound 1	−0.045	0.741 **	−0.259	−0.303	0.591 *			
Compound 2	0.121	0.744 ***	0.119	0.040	0.817 ***	0.586 *		
Sum of compounds 1 and 2	0.090	0.804 ***	0.036	−0.041	0.828 ***	0.735 **	0.980 ***	

0.7 < P < 1.0, strong linear correlation; 0.3 < P < 0.7, significant linear correlation; (P = Pearson’s correlation coefficient). Significance * *p* < 0.05, ** *p* < 0.01, *** *p* < 0.001

## Data Availability

The data presented in this study are available in supplementary material.

## Supplementary Materials

The following are available online, Figure S1: MS chromatogram in negative ionization mode (A); UV chromatogram at 280 nm (B) of extracts of *V. amurensis* roots, Figure S2: ^1^H-and ^13^C-NMR spectra of compound **1** (300 and 75 MHz, CD_3_OD), Figure S3: ^1^H-and ^13^C-NMR spectra of compound **2** (300 and 75 MHz, CD_3_OD), Figure S4: Sigmoidal plot and IC_50_ of positive control, compounds **1** and **2****,** Figure S5: Response surface and contour plots showing the effect of extraction parameters (X1: extraction time, min; X2: MeOH concentration, %; X3: solvent volume, mL). (A) yield; (B) tyrosinase inhibitory activity; (C) compound **1**; (D) compound **2**; (E) sum of compounds **1** and **2**, Table S1. Tyrosinase inhibitory activity of extracts of *V. amurensis* roots, Table S2: ^1^H- and ^13^C NMR data of compounds **1** and **2** in CD_3_OD (δ in ppm), Table S3: Independent variables and levels for response surface methodology, Table S4: Estimated regression coefficient for yield, Table S5: Analysis of variance for yield, Table S6: Estimated regression coefficient for tyrosinase inhibitory activity, Table S7: Analysis of variance for tyrosinase inhibitory activity, Table S8: Estimated regression coefficient for compound **1**, Table S9: Analysis of variance for compound **1**, Table S10: Estimated regression coefficient for compound 2, Table S11. Analysis of variance for compound **2**, Table S12: Estimated regression coefficient for sum of compounds **1** and **2**, Table S13: Analysis of variance for sum of compounds **1** and **2**.

## Sample Availability

Not available.
