# Supplementary material for: Characterization and Optimization of the Tyrosinase Inhibitory Activity of Vitis amurensis Root Using LC-Q-TOF-MS Coupled with a Bioassay and Response Surface Methodology
Source: Molecules. 2021 Jan 16;26(2):446. doi: 10.3390/molecules26020446 (PMC7830106; doi:10.3390/molecules26020446)

Supporting information

Characterization and optimization of the tyrosinase inhibitory activity of *Vitis amurensis* root using LC-Q-TOF-MS coupled with a bioassay and response surface methodology

Kyung-Eon Oh[1,#], Hyeji Shin[1,#], Mi Kyeong Lee[2], Byoungduck Park[3,*], Ki Yong Lee[1,*]

[1] College of Pharmacy, Korea University, Sejong, Republic of Korea; ok1000sa@korea.ac.kr (K-E.O); hjshin90@korea.ac.kr (H.S)

[2] College of Pharmacy, Chungbuk National University, Cheongju 28160, Republic of Korea; mklee@chungbuk.ac.kr (M.K.L)

[3] College of Pharmacy, Keimyung University, Daegu, Republic of Korea

[#] These authors contributed equally to this work

* Correspondence: bdpark@kmu.ac.kr (B.P.); kylee11@korea.ac.kr (K.Y.L.); Tel.: +82-53-580-6653 (B.P.); Tel.: +82-44-860-1623 (K.Y.L)

Legends of Table and Figure

Table S1. Tyrosinase inhibitory activity of extracts of *V. amurensis* roots.

Table S2. ^{1}H- and ^{13}C NMR data of compounds **1** and **2** in CD_3OD (δ in ppm).

Table S3. Independent variables and levels for response surface methodology.

Table S4. Estimated regression coefficient for yield.

Table S5. Analysis of variance for yield.

Table S6. Estimated regression coefficient for tyrosinase inhibitory activity.

Table S7. Analysis of variance for tyrosinase inhibitory activity.

Table S8. Estimated regression coefficient for compound **1**.

Table S9. Analysis of variance for compound **1**.

Table S10. Estimated regression coefficient for compound **2**.

Table S11. Analysis of variance for compound **2**.

Table S12. Estimated regression coefficient for sum of compounds **1** and **2**.

Table S13. Analysis of variance for sum of compounds **1** and **2**.

Figure S1. MS chromatogram in negative ionization mode (A); UV chromatogram at 280 nm (B) of extracts of *V. amurensis* roots.

Figure S2. ^{1}H-and ^{13}C-NMR spectra of compound **1** (300 and 75MHz, CD_3OD)

Figure S3. ^{1}H-and ^{13}C-NMR spectra of compound **2** (300 and 75MHz, CD_3OD)

Figure S4. Sigmoidal plot and IC_{50} of positive control, compounds **1** and **2**

Figure S5. Response surface and contour plots showing the effect of extraction parameters (X1: extraction time, min; X2: MeOH concentration, %; X3: solvent volume, mL). (A) yield; (B) tyrosinase inhibitory activity; (C) compound **1**; (D) compound **2**; (E) sum of compounds **1** and **2**

Table S1. Tyrosinase inhibitory activity of extracts of *V. amurensis* roots.

		**TI activity (%)**[b]
Control		0.0 ± 3.63
Kojic acid[a]		72.5 ± 1.0
80% MeOH extract	1μg/ml	-4.6 ± 1.2
	2.5μg/ml	-1.4 ± 1.0
	5μg/ml	2.3 ± 0.3
	7μg/ml	8.1 ± 0.7***
	10μg/ml	16.1 ± 0.7***
	30μg/ml	61.2 ± 3.5***
	50μg/ml	80.7 ± 0.8***

[a]Positive control, 7.1 μg/ml (50 μM)

[b]Tyrosinase inhibitory activity

*p<0.05, **p<0.01, ***p<0.001 compare with control

Table S2. ^{1}H- and ^{13}C NMR data of compounds **1** and **2** in CD_3OD (δ in ppm).

position	**1**		**2**		Position	**2**	
	δ_H	δ_C	δ_H	δ_C		δ_H	δ_C
1		128.9		133.2	1''		130.9
2	7.00 (d, 8.3)	127.4	7.19 (d, 7.5)	126.4	2''	6.70 (s)	125.3
3	6.61 (d, 8.3)	115.0	6.81 (d, 7.5)	115.1	3''		109.3
4		157.1		157.0	4''		158.8
5	6.61 (d, 8.3)	115.0	6.81 (d, 7.5)	115.1	5''	6.76 (d, 7.1)	118.6
6	7.00 (d, 8.3)	127.4	7.19 (d, 7.5)	126.4	6''	7.04 (d, 7.1)	124.2
7	6.54 (d, 16.6)	122.3	5.41 (d, 7.1)	93.3	7''	6.55 (d, 12.0)	129.1
8	6.80 (d, 16.6)	129.0	4.39 (d, 7.1)	56.5	8''	6.74 (d, 12.0)	122.8
9		135.5		146.4	9''		135.4
10		118.6	6.17 (s)	105.6	10''		103.2
11		161.3		158.7	11''		158.2
12	6.58 (brs)	95.4	6.17 (s)	101.1	12''	6.28 (s)	95.5
13		157.0		158.5	13''		161.4
14	6.19 (brs)	102.9	6.17 (s)	105.6	14''	6.61 (s)	114.7
1'		132.5		131.3	1'''		132.5
2'	7.10 (d, 7.5)	126.8	6.63 (d, 7.4)	126.4	2'''	7.23 (d, 8.1)	126.8
3'	6.72 (d, 7.5)	114.9	6.57 (d, 7.4)	114.6	3'''	6.88 (d, 8.1)	114.9
4'		158.4		156.6	4'''		157.1
5'	6.72 (d, 7.5)	114.9	6.57 (d, 7.4)	114.6	5'''	6.88 (d, 8.1)	114.9
6'	7.10 (d, 7.5)	126.8	6.63 (d, 7.4)	126.4	6'''	7.23 (d, 8.1)	126.8
7'	5.32 (d, 6.5)	93.4	5.47 (d, 5.1)	90.8	7'''	5.37 (d, 4.7)	93.4
8'	4.30 (d, 6.5)	56.9	4.29 (d, 5.1)	51.6	8'''	4.41 (d, 4.7)	56.8
9'		146.0		141.1	9'''		145.9
10'	6.11 (brs)	106.0		105.7	10'''	6.02 (s)	106.1
11'		158.7		159.1	11'''		158.6
12'	6.11 (brs)	100.8	6.32 (s)	95.2	12'''	6.10 (s)	100.9
13'		158.7		161.3	13'''		158.6
14'	6.11 (brs)	106.0	6.13 (s)	118.7	14'''	6.02 (s)	106.1

Table S3. Independent variables and levels for response surface methodology

Independent variables	Coded symbols	Levels		
		-1	0	1
Extraction time (min)	X_1	40	70	100
MeOH concentration (%)	X_2	40	70	100
Solvent volume (mL)	X_3	35	87.5	140

Table S4. Estimated regression coefficient for yield.

Term	Coefficient	SE Coefficient	*T*-value	*P*-value
**Constant**	4.49333	0.3637	12.353	0.000
**Linear**				
X_1 (Time)	0.51625	0.2227	2.318	0.068
X_2 (MeOH)	-0.29125	0.2227	1.308	0.248
X_3 (Volume)	0.90750	0.2227	4.074	0.010
**Square**				
X_1^2	-0.18292	0.3279	-0.558	0.601
X_2^2	-1.31292	0.3279	-4.004	0.010
X_3^2	0.35458	0.3279	1.081	0.329
**Interaction**				
X_1X_2	-0.05750	0.3150	-0.183	0.862
X_1X_3	0.08000	0.3150	0.254	0.810
X_2X_3	-0.10500	0.3150	-0.333	0.752
**R-Sq = 89.4%** **R-Sq(adj) = 70.2%**				

Table S5. Analysis of variance for yield.

Source	DF	Seq SS	Adj SS	MS	*F*-value	*P*-value
**Regression**	9	16.6665	16.66653	1.85184	4.67	0.052
Linear	3	9.3992	9.39918	3.13306	7.89	0.024
Square	3	7.1844	7.18443	2.39481	6.03	0.041
Interaction	3	0.0829	0.08292	0.02764	0.07	0.974
**Residual error**	5	1.9846	1.98464	0.39693		
Lack of fit	3	1.5012	1.50117	0.50039	2.07	0.342
Pure error	2	0.4835	0.48347	0.24173		
**Total**	14	18.6512				

Table S6. Estimated regression coefficient for tyrosinase inhibitory activity.

Term	Coefficient	SE Coefficient	*T*-value	*P*-value
**Constant**	89.0367	2.073	42.961	0.000
**Linear**				
X_1 (Time)	3.7988	1.269	2.993	0.030
X_2 (MeOH)	12.3313	1.269	9.716	0.000
X_3 (Volume)	1.5600	1.269	1.229	0.274
**Square**				
X_1^2	-5.0058	1.868	-2.680	0.044
X_2^2	-8.7608	1.868	-4.690	0.005
X_3^2	-1.9933	1.868	-1.067	0.335
**Interaction**				
X_1X_2	-5.8950	1.795	-3.284	0.022
X_1X_3	0.5225	1.795	0.291	0.783
X_2X_3	-1.9275	1.795	-1.074	0.332
**R-Sq = 96.7% R-Sq(adj) = 90.6%**				

Table S7. Analysis of variance for tyrosinase inhibitory activity.

Source	DF	Seq SS	Adj SS	MS	*F*-value	*P*-value
**Regression**	9	1863.25	1863.25	207.028	16.07	0.003
Linear	3	1351.39	1351.39	450.464	34.96	0.001
Square	3	356.90	356.90	118.967	9.23	0.018
Interaction	3	154.96	154.96	51.652	4.01	0.085
**Residual error**	5	64.43	64.43	12.886		
Lack of fit	3	57.87	57.87	19.290	5.88	0.149
Pure error	2	6.56	6.56	3.280		
**Total**	14	1927.68				

Table S8. Estimated regression coefficient for compound **1**.

Term	Coefficient	SE Coefficient	*T*-value	*P*-value
**Constant**	12.1300	3.213	3.775	0.013
**Linear**				
X_1 (Time)	-0.6350	1.968	-0.323	0.760
X_2 (MeOH)	10.5675	1.968	5.370	0.003
X_3 (Volume)	-3.6875	1.968	-1.874	0.120
**Square**				
X_1^2	-5.9350	2.897	-2.049	0.096
X_2^2	2.1350	2.897	0.737	0.494
X_3^2	0.0800	2.897	0.028	0.979
**Interaction**				
X_1X_2	-1.2800	2.793	-0.460	0.665
X_1X_3	-0.1000	2.783	-0.036	0.973
X_2X_3	-8.7100	2.783	-3.130	0.026
**R-Sq = 90.5% R-Sq(adj) = 73.3%**				

Table S9. Analysis of variance for compound **1**.

**Source**	**DF**	**Seq SS**	**Adj SS**	**MS**	***F*-value**	***P*-value**
**Regression**	9	1470.90	1470.90	163.433	5.28	0.041
Linear	3	1005.38	1005.38	335.128	10.82	0.013
Square	3	155.47	155.47	51.822	1.67	0.287
Interaction	3	310.05	310.05	103.350	3.34	0.114
**Residual error**	5	154.89	154.89	30.979		
Lack of fit	3	153.78	153.78	51.261	92.35	0.011
Pure error	2	1.11	1.11	0.555		
**Total**	14	1625.79				

Table S10. Estimated regression coefficient for compound **2**.

**Term**	**Coefficient**	**SE Coefficient**	***T*-value**	***P*-value**
**Constant**	86.5067	5.263	16.436	0.000
**Linear**				
X_1 (Time)	5.8925	3.223	1.828	0.127
X_2 (MeOH)	36.2450	3.223	11.246	0.000
X_3 (Volume)	5.7875	3.223	1.796	0.133
**Square**				
X_1^2	-31.2371	4.744	-6.584	0.001
X_2^2	-15.7721	4.744	-3.324	0.021
X_3^2	-32.0421	4.744	-6.754	0.001
**Interaction**				
X_1X_2	5.4425	4.558	1.194	0.286
X_1X_3	-3.2775	4.558	-0.719	0.504
X_2X_3	-0.3725	4.558	-0.082	0.938
**R-Sq = 97.8%**	**R-Sq(adj) = 93.9%**			

Table S11. Analysis of variance for compound **2**.

**Source**	**DF**	**Seq SS**	**Adj SS**	**MS**	***F*-value**	***P*-value**
**Regression**	9	18555.7	18555.7	2061.75	24.81	0.001
Linear	3	11055.3	11055.3	3685.11	44.34	0.001
Square	3	7338.4	7338.4	29.43	0.001	
Interaction	3	162.0	162.0	54.00	0.65	0.616
**Residual error**	5	415.5	415.5	83.11		
Lack of fit	3	219.9	219.9	73.30	0.75	0.615
Pure error	2	195.6	195.6	97.82		
**Total**	14	18971.3				

Table S12. Estimated regression coefficient for sum of compounds **1** and **2**.

**Term**	**Coefficient**	**SE Coefficient**	***T*-value**	***P*-value**
**Constant**	98.633	4.793	20.578	0.000
**Linear**				
X_1 (Time)	5.293	2.935	1.792	0.133
X_2 (MeOH)	46.815	2.935	15.949	0.000
X_3 (Volume)	2.096	2.935	0.714	0.507
**Square**				
X_1^2	-37.173	4.321	-8.604	0.000
X_2^2	-13.635	4.321	-3.156	0.025
X_3^2	-31.963	4.321	-7.398	0.001
**Interaction**				
X_1X_2	4.160	4.151	1.002	0.362
X_1X_3	-3.378	4.151	-0.814	0.453
X_2X_3	-9.080	4.151	-2.187	0.080
**R-Sq = 98.7%** **R-Sq(adj) = 96.4%**				

Table S13. Analysis of variance for sum of compounds **1** and **2**.

Source	DF	Seq SS	Adj SS	MS	*F*-value	*P*-value
**Regression**	9	26764.7	26764.7	2973.86	43.15	0.000
Linear	3	17789.5	17789.5	5929.85	86.04	0.000
Square	3	8530.6	8530.6	2843.52	41.26	0.001
Interaction	3	444.6	444.6	148.21	2.15	0.212
**Residual error**	5	344.6	344.6	68.92		
Lack of fit	3	121.0	121.0	40.33	0.36	0.792
Pure error	2	223.6	223.6	111.82		
**Total**	14	27109.4				

Figure S1. MS chromatogram in negative ionization mode (A); UV chromatogram at 280 nm (B) of extracts of *V. amurensis* roots.

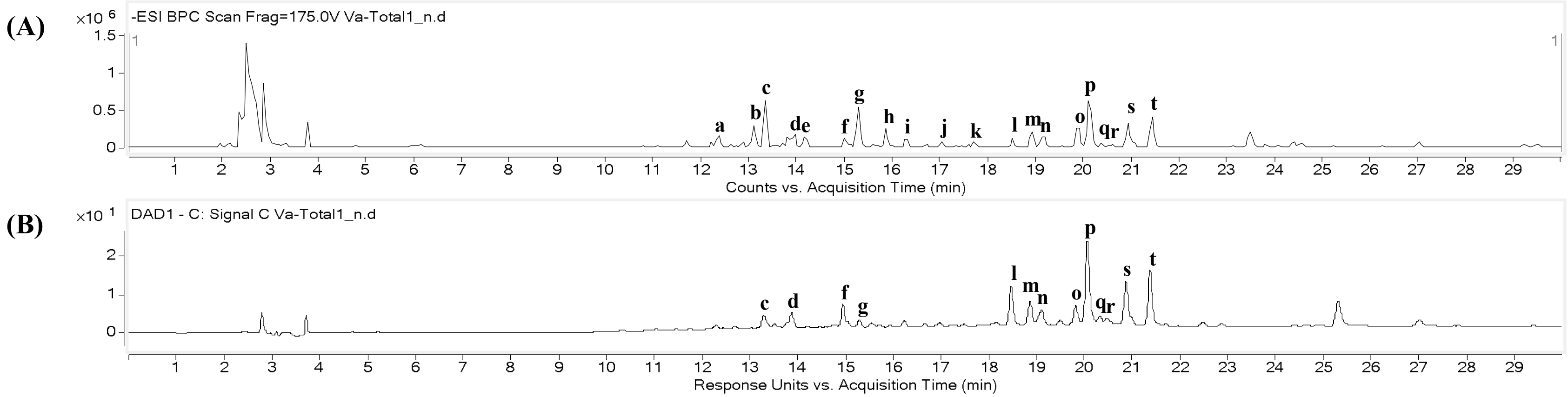

Peak No.	Compounds identification	t_R (mins)	Observed m/z	Calculated m/z	Molecular formula $[M-H]^-$	MS/MS fragments (m/z)	UV (λmax, nm)	Compound No.
a	Catechin	12.389	289.0709	289.0718	$C_{15}H_{13}O_6$	$245[M-C_2H_4O-H]^-$, $203[M-4OH-H_2O-H]^-$	280	
b	Resveratrol glycoside	13.106	377.1814	377.1242	$C_{19}H_{21}O_8$	$331[M-C_2H_6O-H]^-$	280	
c	Epicatechin	13.362	289.0719	289.0718	$C_{15}H_{13}O_6$	$245[M-C_2H_4O-H]^-$, $203[M-4OH-H_2O-H]^-$	280	
d	Resveratroloside	13.977	435.1283	435.1297	$C_{21}H_{23}O_{10}$*	$389[M-H]^-$, $227[M-C_6H_{10}O_5-H]^-$	285	
e	Piceid	15.001	389.1239	389.1242	$C_{20}H_{21}O_8$	$227[M-C_6H_{10}O_5-H]^-$, $185[M-C_7H_6O-4OH-CH_2O-H]^-$	307, 322	
f	Resveratrol trimer	15.309	723.5033	723.5053	$C_{41}H_{71}O_{10}$*	$677[M-H]^-$		
g	Vitisifuran	15.872	949.6700	949.2502	$C_{56}H_{40}O_{12}$*	$677[M-C_{14}H_{10}O_3-H]^-$,$451[M-C_{28}H_{20}O_6-H]^-$		
h	Ampelopsin A	16.333	469.1291	469.1293	$C_{28}H_{21}O_7$	$375[M-C_6H_6O-H]^-$, $451[M-H_2O-H]^-$	280	
i	Piceatannol	16.743	243.0654	243.0663	$C_{14}H_{11}O_4$	$201[M-C_2H_2O-H]^-$, $175[M-4OH-H]^-$	282, 324	
j	Ampelopsin F	17.05	453.1338	453.1344	$C_{28}H_{21}O_6$	$359[M-C_6H_6O-H]^-$	280	
k	Syringetin-3-O-β-D-glucopyranoside	17.716	507.2073	507.2083	$C_{22}H_{35}O_{13}$	$461[M-CHO-OH-H]^-$,$293[M-C_6H_{11}O_5-3OH-H]^-$		
l	Resveratrol	18.535	227.0704	227.0714	$C_{14}H_{11}O_3$	$185[M-C_2H_2O-H]^-$, $143[M-C_4H_4O_2-H]^-$	220	
m	Ampelopsin C	18.945	679.1965	679.1974	$C_{42}H_{31}O_9$	$585[M-C_6H_6O-H]^-$	302, 322	
n	Hopeaphenol/Isohopeaphenol	19.150	905.259	905.2604	$C_{56}H_{41}O_{12}$	$358[M-C_{28}H_{22}O_6-C_6H_6O-H]^-$, $811[M-C_6H_6O-H]^-$	284, 220	
o	Cis-ε-viniferin	19.918	453.1341	453.1344	$C_{28}H_{21}O_6$	$347[M- M-C_7H_6O-H]^-$		
p	Trans-ε-viniferin	20.123	453.1348	453.1344	$C_{28}H_{21}O_6$	$359[M-C_6H_6O-H]^-$, $347[M-C_7H_6O-H]^-$, $225[M-C_8H_7O-C_6H_5O-H]$	284, 330	1
q	Vitisin A (γ2-viniferin)	20.247	905.2573	905.2604	$C_{56}H_{41}O_{12}$	$811[M-C_6H_6O-H]^-$, $359[M-C_{28}H_{21}O_6-C_6H_6O-H]^-$	285, 330,	
r	Miyabenol C	20.635	679.195	679.1974	$C_{42}H_{31}O_9$	$585[M-C_6H_6O-H]^-$, $478[M-C_6H_5O-C_6H_5O_2-H]^-$	285, 330	
s	Resveratrol tetramer	20.943	905.2589	905.2604	$C_{56}H_{41}O_{12}$	$799\ [M-C_7H_6O-H]^-$, $359\ [M-C_{28}H_{21}O_6-C_6H_6O-H]^-$		
t	Vitisin B (γ-viniferin)	21.455	905.2592	905.2604	$C_{56}H_{41}O_{12}$	$811[M-C_6H_6O-H]^-$	285, 313	2

* $[M+HCOO^-]^-$

Figure S2. ^{1}H-and ^{13}C-NMR spectra of compound **1** (300 and 75MHz, CD_3OD)

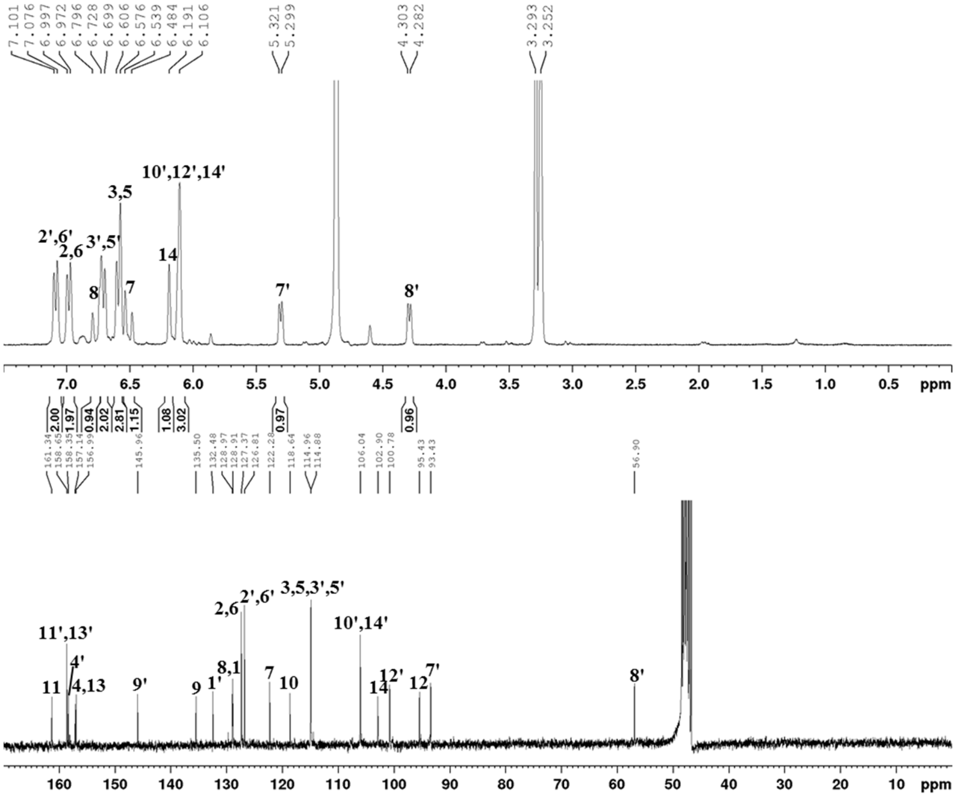

Figure S3. ^{1}H-and ^{13}C-NMR spectra of compound **2** (300 and 75MHz, CD_3OD)

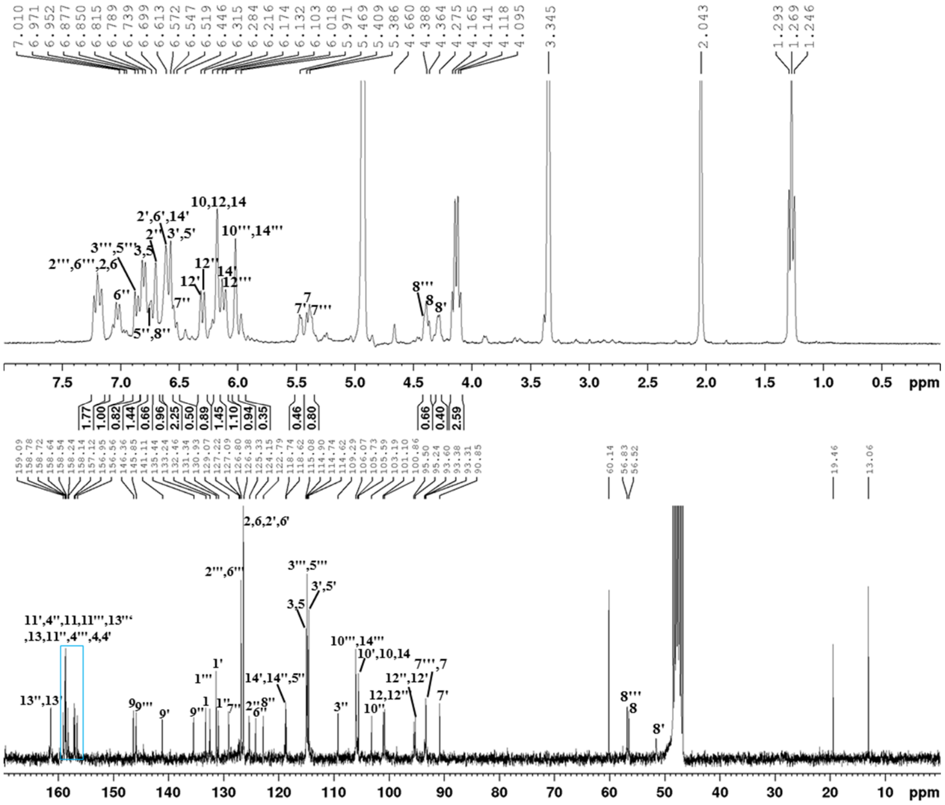

Figure S4. Sigmoidal plot and IC_{50} of positive control, compounds **1** and **2**

(A)

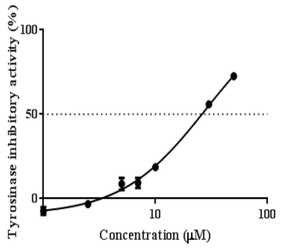

(B)

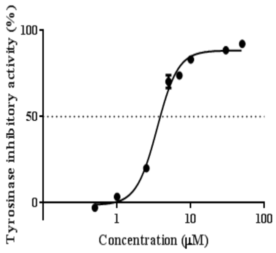

(C)

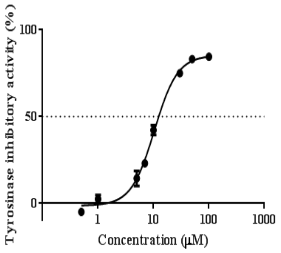

(A) Positive control, kojic acid (IC_{50} 27.09 ± 2.8 μM); (B) compound **1**, ε-viniferin (IC_{50} 3.51 ± 0.1 μM);

(C) compound **2**, vitisin B (IC_{50} 10.74 ± 1.3 μM)

Figure S5. Response surface and contour plots showing the effect of extraction parameters (X1: extraction time, min; X2: MeOH concentration, %; X3: solvent volume, mL). (A) yield; (B) tyrosinase inhibitory activity; (C) compound **1**; (D) compound **2**; (E) sum of compounds **1** and **2**

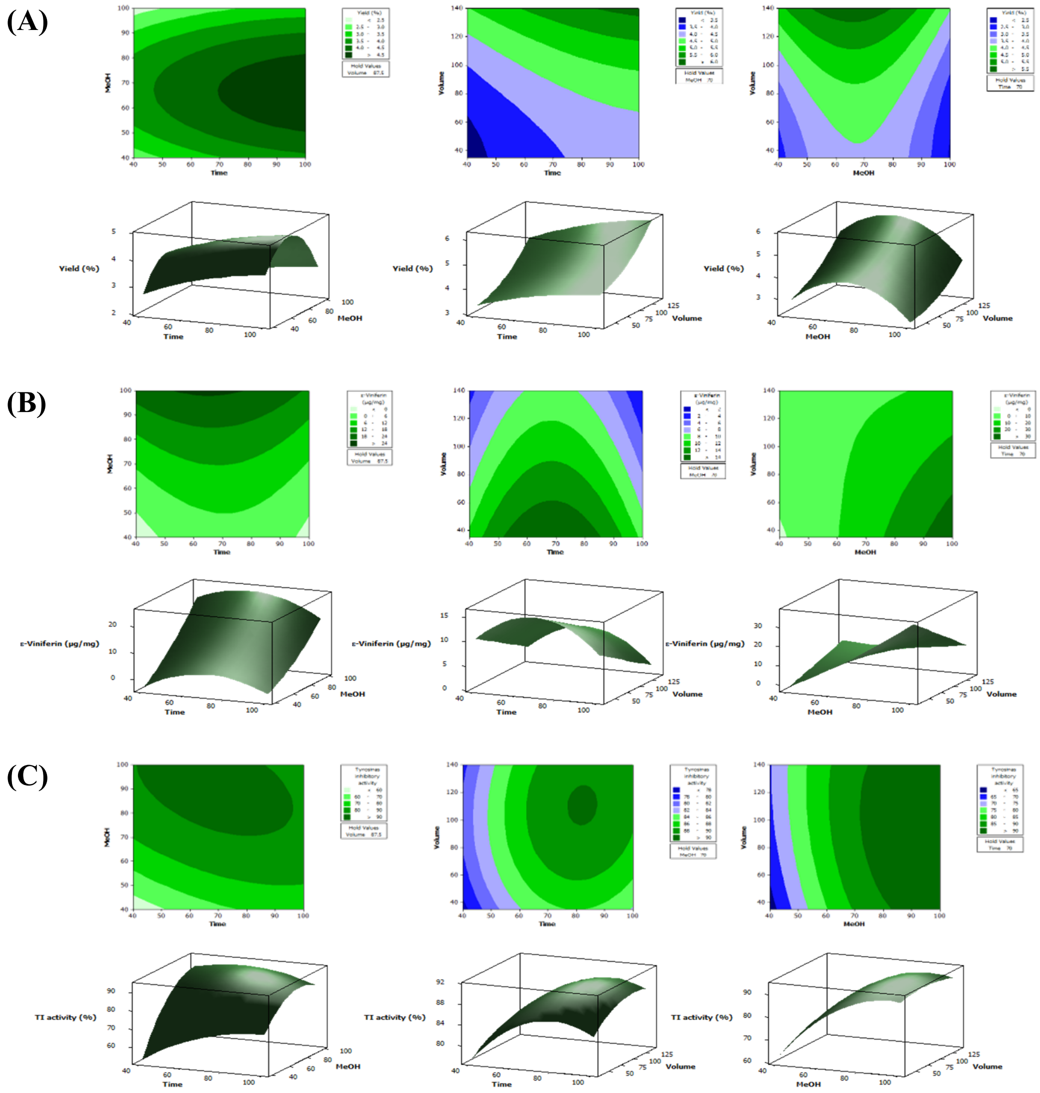

Figure S5. Continued

(D)

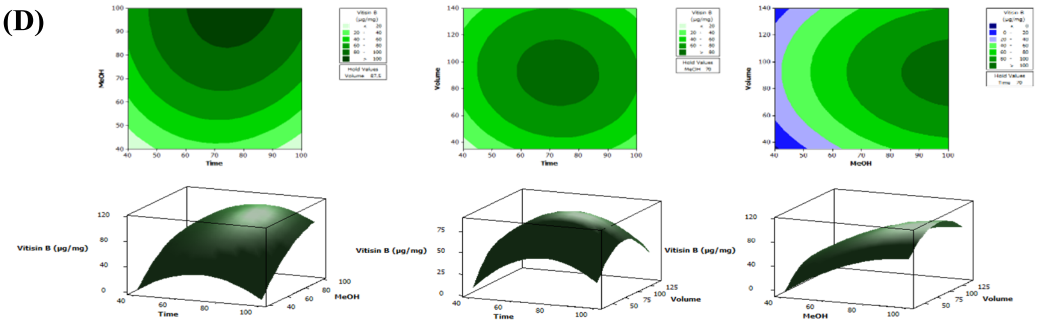

(E)

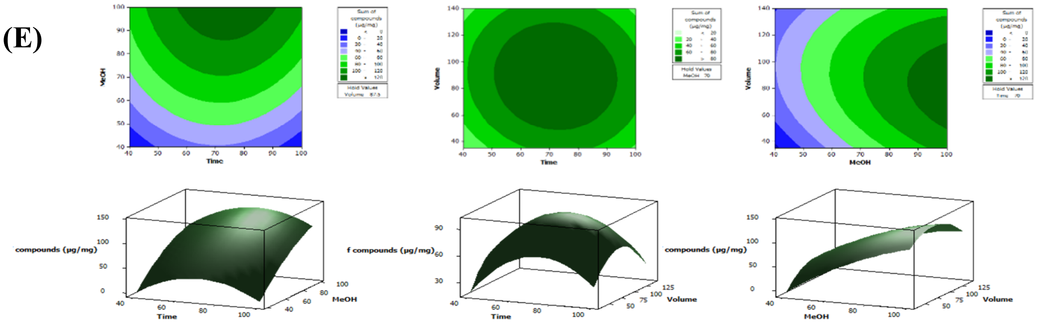

Supplement: Supplementary file 1 [file molecules-26-00446-s001.pdf]
